# Effect of androgen deprivation therapy on cardiovascular function in Chinese patients with advanced prostate cancer: a prospective cohort study

**DOI:** 10.1038/s41598-020-75139-w

**Published:** 2020-10-22

**Authors:** Chi-fai Ng, Peter K. F. Chiu, Chi-hang Yee, Becky S. Y. Lau, Steven C. H. Leung, Jeremy Y. C. Teoh

**Affiliations:** SH Ho Urology Centre, Department of Surgery, The Chinese University of Hong Kong, Shatin, Hong Kong

**Keywords:** Prostate cancer, Dyslipidaemias, Coronary artery disease and stable angina, Metabolic syndrome

## Abstract

Androgen deprivation therapy (ADT) is the standard treatment for advanced prostate cancer, but its effect on cardiovascular and metabolic function in Asian patients is still inconclusive. We prospectively assess the effects of ADT on 36 patients with advanced prostate cancer, with reference to another 24 prostate cancer patients not requiring ADT, for 2 years. Patients’ anthropometric, metabolic and vascular parameters were assessed every six-monthly. The baseline parameters of the two groups were comparable. There was a significant negative effect of the usage of ADT on the changes in BMI (p = 0.020), waist to hip ratio (p = 0.005), body fat percentage (p = 0.012), and high-density-lipoprotein (p = 0.012). ADT-patients were 4.9 times more likely to have metabolic syndrome at 24 months. (CI 0.889–27.193, p = 0.068). The Framingham risk score (p = 0.018) and pulse-wave-velocity (p = 0.024) for ADT-group were also significantly higher than controls, which signified increase in cardiovascular risk. Although there was no statistically significant difference in ischemic cardiovascular events between two groups, a trend for more events in ADT-group was observed. Therefore, Asian patients have increased cardiovascular and metabolic risks after being treated with ADT for two years. Appropriate counselling and monitoring of associated complications with ADT are essential.

## Introduction

Since the ground-breaking work of Charles Huggins on the role of androgen deprivation therapy (ADT) in prostate cancer, it has become one of the key players in the management of prostate cancer (PCa), particularly in metastatic disease^1^. Even in castration resistant stage, ADT is still considered the backbone therapy for most patients^1^. Currently, beside the traditional bilateral orchidectomy (surgical castration), there were also luterizing hormone releasing hormone agonist (LHRH agonist) and antagonist (LHRH antagonist) available as options for medical castration^1^. The choice of treatment would be depend on the clinical conditions (need of rapid testosterone suppression), financial implications, patient preference etc.

With the advancements in clinical care, the overall survival of prostate cancer patients has prolonged^2^. But there is also increasing concern about potential long-term side effects and even mortality related to ADT in patients with prostate cancer. In some early single-arm prospective studies, ADT were found to be associated with increased body weight and body fat^3^, reduced insulin sensitivity and increased arterial stiffness^4^. These concerns were further supported by observational studies, which showed that patients who received ADT had increased incidence of diabetes, myocardial infarction and even cardiac mortality^5,6^. Unfortunately these results were inconsistent. Some prospective randomized clinical studies reported that ADT were not associated with increase in cardiovascular risk^7^. Despite these controversies, a joint statement was issued by the American Urological Association, the American Society for Radiation Oncology and the American Heart Association to raise physicians’ awareness about the potential association between ADT and cardiovascular disease^8^. Nevertheless, the evidence is still inconclusive even in latest meta-analyses^9^.

The situation in Asia is also controversial, as information on ADT is scanty. Large-scale prospective randomized studies on prostate cancer in Asia are lacking. Studies related to ADT were mainly observational or retrospective in nature. The earliest information was from the J-Cap registry study, which showed that the incidence of cardiovascular events in ADT patients was similar to that of the general population^10^. Thereafter, several groups reported investigations regarding this topic, with some showing increased cardiovascular risk in patients managed with ADT^11,12^, while others suggesting otherwise^13–15^. Therefore, there is a need for further studies to assess the effect of ADT on prostate cancer patients, including the fundamental effect on body compositions, cardiovascular related metabolic changes and cardiovascular risks.

## Methods

This was a prospective cohort study. The objective of this study is to assess the cardiovascular and metabolic effects of ADT in Chinese patients who were diagnosed to have PCa. The study was approved by the institutional ethics committee (Joint Chinese University of Hong Kong-New Territories East Cluster Clinical Research Ethics Committee) and conducted according to the principles of the Helsinki Declaration on human experimentation. Adult Chinese patients with histological diagnosis of PCa whom decided for long term ADT monotherapy were prospectively recruited for the study in our centre. ADT could be in the form of bilateral orchidectomy, luteinizing hormone-releasing hormone (LHRH) agonists or LHRH antagonist. Maximal androgen blockage was not our usual practice, additional short course of androgen receptor blocker was only used as flare prevention in some patients receiving LHRH agonist. Written informed consent was obtained from all subjects.

Additional age-matched Chinese patients with the diagnosis of PCa, who did not receive ADT treatment or other active treatment, were recruited as control arm. These patients were diagnosed to have localized disease and had undergone radical surgery or managed conservatively for their cancer. If the patient was not able to provide consent or comply with the follow-up arrangement, or if based on clinical judgement that the life expectancy of the patient was limited, the patient would be excluded from the study.

### Study protocol

Written informed consent was obtained from all subjects. All patients then underwent a series of clinical, biochemical and vascular function assessment at baseline, followed by 6-monthly assessment for two years to determine their metabolic and cardiovascular status.

#### Clinical assessment

Anthropometric parameters including patients’ height, body weight, BMI, waist and hip circumference were measured. Waist circumference was measured at the level of umbilicus. Hip circumference was defined as the maximum circumference of the buttock. A skinfold caliper was used for the measurement of skinfold thickness for the estimation of the total amount of body fat. Four areas of the body, biceps (front side of the mid-upper arm), triceps (back side of the mid-upper arm), shoulder blade (just below the subscapular) and supra-iliac (just above the iliac crest) were measured. The sum of the four measurements was then used to estimate the body fat percentage using the Durnin and Womersley formula^16^. All the above measurements were performed when the patient stood relaxingly in a warm examination room with privacy. Blood pressure and pulse were measured twice with 5-min interval by an automated machine after resting for 5 min. During each follow-up, besides repeating these measurements, cardiovascular events of the patients were recorded.

#### Laboratory

Blood samples were collected from patients after overnight fasting for the measurements of sugar level, total triglycerides (TG), total cholesterol (TC), low density lipoprotein cholesterol (LDL-C), high density lipoprotein cholesterol (HDL-C) and complete blood picture (including haemoglobin level). Testosterone level was also measured to confirm the castration in patients receiving ADT.

#### Vascular function assessment

In this study, the central and peripheral arterial conditions were assessed by brachial-ankle pulse wave velocity (baPWV) and Ankle brachial index (ABI), respectively. Brachial-ankle pulse wave velocity is an approach to assess central arterial stiffness, which is well-recognized to be associated with cardiovascular disease^17,18^. The system involved four separate cuffs applied to the four limbs and automatic measurement of the blood pressure and pulse wave in the limbs. Combined with the information on body height, it will provide the baPWV of the measured individual. The faster the baPWV, the stiffer the central blood vessel, and higher cardiovascular risk.

Ankle brachial index (ABI) is an index for the assessment of vascular occlusion due to peripheral vascular disease. It is defined as the ratio of the blood pressure of the lower limb, as measured at the ankle, to the blood pressure of the upper limb, as measured at the upper arm (brachial artery). The lower the ratio, i.e. greater difference between the upper and lower limb, the more severe arterial occlusion by peripheral arterial disease.

In this study, both baPWV and ABI were assessed by the Vascular Profiler-1000 machine (Omron, Kyoto, Japan) using the oscillometric cuff technique. Patient was asked to rest in supine position in a quiet and warm environment for at least 10 min. The 4 measuring cuffs would be applied to both arms and ankles, respectively. The machine would then automatically measure the baPWV and ABI. After inputting the height of the patient, baPWV was calculated automatically between the ankle and brachial pulse waveform for both sides. The average of the two sides’ readings would be used for the assessment of baPWV of the subjects in our study.

#### Overall assessment

As many parameters were measured in our study, we used the Framingham Risk Score as the primary endpoint to assess the overall cardiovascular risk of the patients during the study period. The Framingham Risk Score is a gender-specific algorithm based on multiple cardiovascular risk factors including age, smoking status, blood pressure level, serum total cholesterol and HDL-cholesterol level for the estimation of the 10-year risk of developing cardiovascular disease^19^. We also applied the National Cholesterol Education Program (NCEP)/Adult Treatment Panel III (ATPIII) guidelines to assess whether the patients had increased risk of developing metabolic syndromes after ADT treatment^20^.

### Statistical analysis

All data were reported as mean ± SD unless otherwise specified. Student’s t test and Fisher’s exact test were used for the comparison between ADT and control groups. Two-way mixed ANOVA was performed to evaluate if any change in the outcome variables is the result of the interaction between usage of ADT and time. All tests were two-sided, with significance set at 5%. Statistical analyses were performed by SPSS (Chicago, IL).

### Take home message

Androgen deprivation therapy in advanced prostate cancer in Chinese are associated with increased cardiovascular and metabolic risks. Appropriate counselling and monitoring of these complications are essential.

## Results

From July 2011 to January 2016, 36 patients with prostate cancer managed with primary ADT alone were recruited to the study. Amongst them, 20 patients had metastatic disease and the other 16 patients were diagnosed with locally advanced disease. The number of patients chose bilateral orchidectomy, LHRH agonist and LHRH antagonist as the initial ADT were 18, 11 and 7, respectively (Table 1). The choice of medical or surgical castration depended on patient preference and also financial implication, as the cost of medical castration was not reimbursed. Moreover, LHRH antagonist was only available in 2013 in our area and was mainly used in patients with high volume metastasis (for rapid testosterone suppression) and increased cardiovascular risk. Due to the frequent injection schedule (monthly) and higher cost for LHRH antagonist (degarelix), some patients initially started on LHRH antagonist changed to use 3-monthly LHRH agonist during their course of treatment. Another 24 patients with localized prostate cancer were also recruited as control for the study. Eighteen of them had radical prostatectomy performed while the other six were under watchful waiting or active surveillance. The average time between diagnosis and recruitment for the ADT arm and the control arm was 5.5 and 38.5 months, respectively.

**Table 1:** The choice of androgen deprivation therapy for the 36 patients.

Choice of ADT	Drugs used	Number of patients	Total patients in each category of ADT
Bilateral Orchidectomy		18	18
LHRH agonist	Leuprorelin*	9	11
	Triptorelin*	2	
LHRH antagonist	Degarelix alone	2	7
	Degarelix for 6 months and then Leuprorelin*	4	
	Degarelix for 6 months and then Triptorelin*	1	
		Total number of patients	36

*ADT* Androgen deprivation therapy, *LHRH agonist* luterizing hormone releasing hormone agonist, *LHRH antagonist* luterizing hormone releasing hormone antagonist.

All LHRH agonist used were 3-monthly formulation.

*P < 0.05.

The baseline characteristics and blood parameters of the patients were listed in Tables 2 and 3. The mean age of the whole group was 74.1 (range 52 to 90) years old, and there was no significant difference between the two groups. Most of the patients had baseline cardiovascular and metabolic diseases. The most common ones were hypertension, hyperlipidaemia and diabetes. About 10% of patients in each arm had a history of cerebrovascular diseases. Four patients (11.1%) in the ADT arm had a history of ischemic heart disease. However, there was no significant difference between the two groups for these medical problems. Similarly, there was no difference in the smoking status between the two groups. The baseline body weight, body height, body mass index (BMI), waist circumference, hip circumference, waist-hip ratio and body fat composition between the two groups were also similar. Fourteen patients (38.89%) in the ADT arm and seven patients (29.17%) in the control arm had metabolic syndrome (p = 0.439). The baseline Framingham risk score for the ADT group and control group were 0.42 and 0.40 respectively (p = 0.720). For vascular assessment, the baseline ankle-brachial index (ABI) for ADT and control group were 1.03 and 1.13 (p = 0.008). The baseline pulse wave velocity (PWV) for ADT and control group were 1766 cm/s and 1779 cm/s (p = 0.889).

**Table 2:** Baseline characteristics.

	Control (n = 24)		ADT (n = 36)		p-value
	Mean/count	SD/percentage	Mean/count	SD/percentage	
**Age at screen**	73.25	4.09	74.67	9.11	0.417
**Duration of diagnosis (month)**	49.88	33.80	9.94	17.04	< 0.001*
**Duration of ADT (day)**	NA	NA	61.47	35.32	0.591
**Mode of ADT**					
BSO	NA	NA	18	50.00%	
LHRH agonist	NA	NA	11	30.6%	
LHRH antagonist	NA	NA	7	19.4%	
**Past medical history**					
Hyperlipidemia	8	33.33%	11	30.56%	0.821
Hypertension	16	66.67%	22	61.11%	0.662
Diabetes mellitus	5	20.83%	10	27.78%	0.543
Ischemic heart disease	0	0.00%	4	11.11%	0.143
Cerebrovascular accident	2	8.33%	4	11.11%	1.000
**Smoking status**					0.755
Non-smoker	13	54.17%	16	44.44%	
Ex-smoker	8	33.33%	15	41.67%	
Chronic smoker	3	12.50%	5	13.89%	
**Baseline ECOG status**					0.382
Fully active	22	91.67%	29	80.56%	
Light work	2	8.33%	5	13.89%	
Ambulatory but no work	0	0.00%	2	5.56%	
**Baseline health assessment**					
Body weight (kg)	64.57	7.37	64.62	10.14	0.983
Body height (m)	1.67	0.06	1.65	0.06	0.113
Body mass index (BMI)	23.15	2.83	23.75	3.08	0.448
Waist circumference (cm)	89.25	8.06	92.14	9.87	0.237
Hip circumference (cm)	94.10	5.87	96.24	6.74	0.211
Waist to hip ratio	0.95	0.06	0.96	0.06	0.604
Body Fat (%)	28.32	3.60	29.89	5.49	0.186
**Baseline Framingham risk**	39.79%	19.86%	41.74%	20.94%	0.720
**Baseline vascular assessment**					
Systolic blood pressure	143.40	18.48	161.32	123.33	0.484
Diastolic blood pressure	84.13	10.59	74.61	9.55	0.001*
Ankle-brachial index (ABI)	1.13	0.10	1.03	0.15	0.008
Pulse wave velocity (PWV)	17.79	2.70	17.66	4.30	0.889
**Metabolic syndrome**	7	29.17%	14	38.89%	0.439

**Table 3:** Between group comparison.

	Baseline					Month 24				
	Control (n = 24)		ADT (n = 36)		p-value^a^	Control (n = 23)		ADT (n = 24)		p-value
	Mean	SD	Mean	SD		Mean	SD	Mean	SD	
**Health assessment**										
Body mass index (BMI)	23.15	2.83	23.75	3.08	0.448	22.90	2.87	24.37	2.97	0.020*
Waist to hip ratio	0.95	0.06	0.96	0.06	0.604	0.93	0.07	0.98	0.06	0.018*
Body fat (%)	28.32	3.60	29.89	5.49	0.186	28.12	3.17	32.44	3.65	0.012*
**Laboratory evaluation**										
Total cholesterol	5.17	0.85	4.96	0.86	0.353	4.81	0.87	4.46	0.85	0.791
Triglyceride	1.35	0.63	1.58	0.89	0.286	1.18	0.43	1.56	0.83	0.673
High density lipoprotein (HDL)	1.54	0.39	1.53	0.41	0.948	1.60	0.43	1.26	0.32	0.012*
Low density lipoprotein (LDL)	3.02	0.80	2.75	0.75	0.183	2.67	0.81	2.49	0.74	0.779
Fasting blood glucose level	5.86	1.02	6.39	1.99	0.237	6.00	0.97	6.74	2.34	0.989
Glycosylated hemoglobin level (HbA1C)	6.14	0.74	6.15	0.78	0.966	6.13	0.69	6.50	1.71	0.491
**Framingham risk**	39.79%	19.86%	41.74%	20.94%	0.720	37.67%	19.93%	47.24%	22.86%	0.018*
**Vascular assessment**										
Systolic blood pressure	143.40	18.48	161.32	123.33	0.484	138.41	13.25	144.44	24.61	0.161
Diastolic blood pressure	84.13	10.59	74.61	9.55	0.001*	80.33	8.85	73.58	11.10	0.148
Ankle-brachial index (ABI)	1.13	0.10	1.03	0.15	0.008	1.12	0.12	1.01	0.14	0.602
Pulse wave velocity (PWV)	17.79	2.70	17.66	4.30	0.889	18.21	3.75	20.52	6.34	0.024*

**Figure 1:**
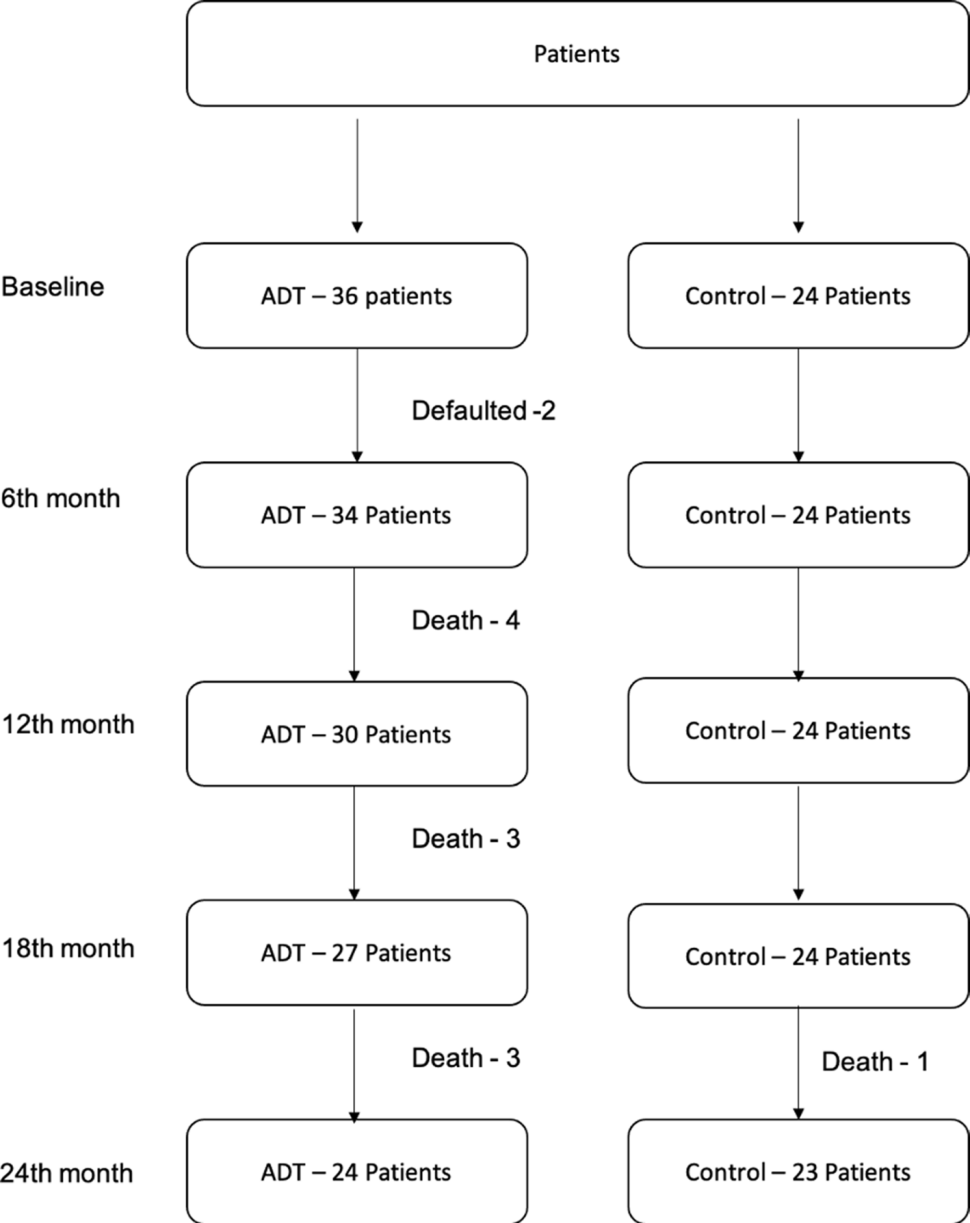
Flowchart of patient follow-up information.

There were two patients in the ADT group who withdrew from the study due to personal reasons. Ten ADT patients and one control patient died before 24 months. The causes of death in the ADT group included progression of prostate cancer (6, 16.67%), right cerebellar haemorrhage (1, 2.78%), sepsis (2, 5.56%), and brain stem glioma (1, 2.78%). One patient in the control arm died of ischemic stroke. In summary, there were 24 ADT patients and 23 control patients who completed the 2-year assessment for analysis. (Fig. 1).

There was a significant interaction between usage of ADT and time in explaining BMI (F_1, 44_ = 5.87, p = 0.020), waist to hip ratio (F_1, 45_ = 5.99, p = 0.018) and body fat percentage (F_1, 44_ = 6.92, p = 0.012) (Table 3). The BMI for ADT group and control group changed from baseline 23.75 ± 3.08 and 23.15 ± 2.83 to 24.37 ± 2.97 and 22.90 ± 2.87, respectively. The waist to hip ratio for ADT group and control group changed from baseline 0.96 ± 0.06 and 0.95 ± 0.06 to 0.98 ± 0.06 and 0.93 ± 0.07 respectively. The body fat percentage for ADT group and control group changed from baseline 29.89 ± 5.49 and 28.32 ± 3.60 to 32.44 ± 3.65 and 28.12 ± 3.17 respectively. These changes suggested patients who received ADT had increased risk of central obesity.

There was a statistically significant interaction between usage of ADT and time in explaining high density lipoprotein (HDL) (Table 3). The HDL for ADT group and control group changed from baseline 1.53 ± 0.41 and 1.54 ± 0.39 to 1.26 ± 0.32 and 1.60 ± 0.43, respectively. On the other hand, there was no significant difference between the two groups in 24-month change in total cholesterol (F_1, 43_ = 0.07, p = 0.791), triglyceride (F_1, 43_ = 0.18, p = 0.673), low density lipoprotein (LDL) (F_1, 43_ = 0.08, p = 0.779), fasting blood glucose (F_1, 43_ < 0.01, p = 0.989) and glycosylated haemoglobin level (HbA1C) (F_1, 32_ = 0.49, p = 0.491). Meanwhile, patients in the ADT arm were 4.918 times more likely to have metabolic syndrome at 24 months compared to control group after adjusting for the baseline (CI 0.889, 27.193, p = 0.068).

**Figure 2:**
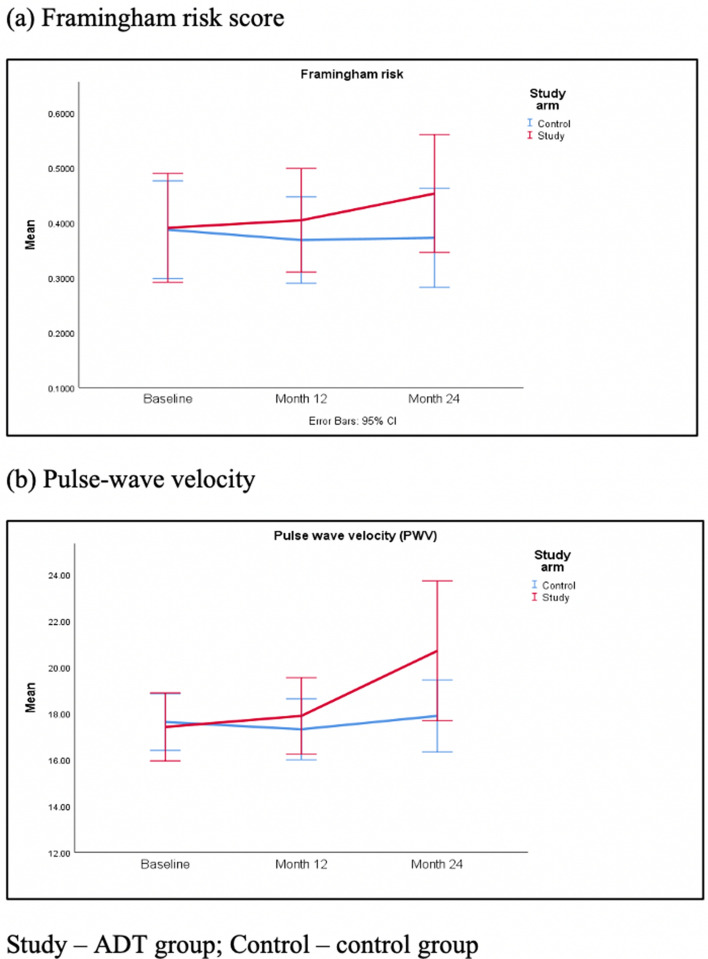
Changes in (**a**) Framingham risk score and (**b**) pulse-wave velocity over time for the two groups. *Study* ADT group, *Control* control group.

The Framingham risk score for ADT group and control group changed from baseline of 41.74% ± 20.94% and 39.79% ± 19.86% to 47.24% ± 22.86% and 37.67% ± 19.93% respectively (Fig. 2a). The results showed that the change in Framingham risk score was significantly different for the two groups (F_1, 43_ = 6.04, p = 0.018).

Vascular assessment showed that there was a significant interaction between usage of ADT and time in explaining pulse wave velocity (PWV) (F_1, 41_ = 5.51, p = 0.024). The PWV for ADT group and control group changed from baseline 17.66 ± 4.30 and 17.79 ± 2.70 to 20.52 ± 6.34 and 18.21 ± 3.75, respectively (Fig. 2b). There was no significant difference between the two groups in 24-month change in systolic blood pressure (F_1, 45_ = 2.03, p = 0.161), diastolic blood pressure (F_1, 45_ = 2.17, p = 0.148) and ankle-brachial index (ABI) (F_1, 42_ = 0.28, p = 0.602).

There were three (8.33%) patients in the ADT group and one (4.17%) patient in the control group who developed ischemic stroke during the 2 year follow-up. Another 3 (8.33%) patients in the ADT group had new onset ischemic heart disease diagnosed during the study period. However, there was no statistically significant difference in ischemic cardiovascular events between the two groups (p = 0.330).

## Discussion

In this prospective comparative study, prostate cancer patients who received ADT had a significant increase in body weight, body fat and central obesity than those without ADT. They also had increased chance of developing metabolic syndrome, higher Framingham risk score and increased pulse wave velocity at the end of 2-year follow-up. All these information points towards that ADT would increase cardiovascular risk and metabolic syndrome. It also led to increase in arterial stiffness and atherosclerosis.

Despite publications on multiple studies and meta-analyses, the effect of ADT on patients was still controversial. In general, meta-analysis of observational studies supported a positive relationship between increased cardiovascular risk and ADT, while the results from randomized studies were negative^9^. Hu et al. proposed that the reason for the conflicting outcomes might be related to the study designs^9^. For observational studies, there may be problems related to confounding factors, treatment adherence and outcome reporting bias. Similarly for randomized studies, underpower of studies, inadequate follow-up period, and selection bias, etc. might contribute to the negative findings. Nevertheless, there is a general consensus that there is an association between ADT usage and cardiovascular risk^21^.

While most of the observational studies on Caucasian population indicated that ADT would increase the cardiovascular risk of patients^9,22^, the results for Asians suggested otherwise. In several observational studies based on national insurance databases in different populations, the results did not show an increased cardiovascular risk in Asian patients receiving ADT^13–15^. The reason for this discrepancy was uncertain. It might be related to the study sample size, racial difference in response to ADT, the overall lower baseline cardiovascular risk of Asian population, and underdiagnosis of cardiovascular events, etc. Nevertheless, this observation reminded us to be cautious in applying findings from Caucasian studies to Asian population.

Therefore, we performed this study to try to bridge the current knowledge gap, the insufficient information about the effect of ADT on Asian population. We planned to overcome some of the pitfalls in previous studies, such as no control arm and short follow-up period^3,4^. While it would be unethical to randomize patients for ADT or not, we included a comparable control arm with prostate cancer patients who did not require ADT. We also included physical, blood and vascular assessments for our patients, with follow-up for up to two years, which was much longer than similar studies^3,4^. While we could continue for even longer follow-up, the dropout rate for ADT arm would be high due to the nature of underlying disease. Six patients in the ADT group developed ischemic cardiovascular events, compared to only one patient in the control group. However, due to the small sample size, this was not statistically significant. Nevertheless, our data suggested that there was a negative effect of ADT on cardiovascular risk and metabolic parameters in our population.

Development and progression of atherosclerosis is believed to be one of the underlying mechanisms related to the increase in cardiovascular risk in patients receiving ADT^23^. In a previous Caucasian study, the usage of ADT was shown to result in significant increase in pulse wave velocity after 3 months of therapy, which is a recognized indicator for major vessels stiffness, and in turn atherosclerotic changes^4,24,25^. However, in a report from Japanese group, there was no significant overall increase in pulse wave velocity after six months of ADT^26^. Our results provided the longest prospective data, up to two years, on the effect of ADT on pulse wave velocity, which further support the initial findings from Caucasian studies. The difference in the results between our study and Oka et al. could be due to study duration, assessment method, or patients’ background. Again, further studies to investigate this area are needed in order to have a better understanding of the situation.

The relative small sample size and single centre experience might limit the generalizability of our findings to other populations. As we aimed to have longer follow-up for our patients, we recruited mainly patients with relatively good performance status in the study. Unfortunately, despite careful selection, only roughly two-thirds of the patients with ADT could survive to the end of the two-year follow-up. Moreover, the repeated assessments had also made some patients hesitate to participate in our study. All these contributed to the slow recruitment rate of the study. In addition, some patients have used LHRH antagonist for treatment, which might have potential cardiovascular protective effect^27^, and hence might affecting the overall incidence of cardiovascular events. Therefore, the small sample size of our study might not be able to detect any significant difference in clinical cardiovascular events, including assessing the effect of different ADT approaches on the outcomes. Despite these, our results had showed convincing evidence of the metabolic and cardiovascular effects of ADT in our patients.

Currently, there are other studies in Asia that are trying to provide more information about the effect of ADT in prostate cancer patients. For example, the real-life evaluation of the effect of ADT in prostate cancer patients in Asia (READT Asia Study) (Clinical trials registration NCT03703778) is a multi-nation prospective study to assess the effect of ADT in prostate cancer patients. The initial result showed that there was a high prevalence of cardiovascular risk factors in Asian prostate cancer patients^28^. Hopefully the study will provide more information on the effects of ADT in Asian patients.

Nevertheless, the result of our study would support the incorporation of additional measures in managing patients treated with ADT to minimize the potential metabolic and cardiovascular harm to patients. The recommended ABCDE approach should be introduced to all patients^29^. These include awareness of the condition and patient education on diet, smoking and exercise, careful monitoring of blood pressure, blood sugar and lipid level for patients, appropriate patient referral to cardiological assessment and usage of pharmacological agents, including aspirin, for high risk patients. For patients with high cardiovascular risk, the usage of LHRH antagonist had shown to have less cardiovascular complications when compared to LHRH agonist^27,30^. All these measures would help to improve the overall standard of care and survival of patients. Currently, there are also studies exploring the use of novel alternate treatments for managing advanced prostate cancer^31,32^. However, most of the these treatments are still in investigational stage, and further studies are needed to assess their roles in clinical management, and also whether they could replace ADT as the primary therapy for patients.
